# Supplementary figures and images for: A local ATR-dependent checkpoint pathway is activated by a site-specific replication fork block in human cells
Source: eLife. 2023 Aug 30;12:RP87357. doi: 10.7554/eLife.87357 (PMC10468204; doi:10.7554/eLife.87357)

Figure 2 - Figure Supplement 1-source data

B)

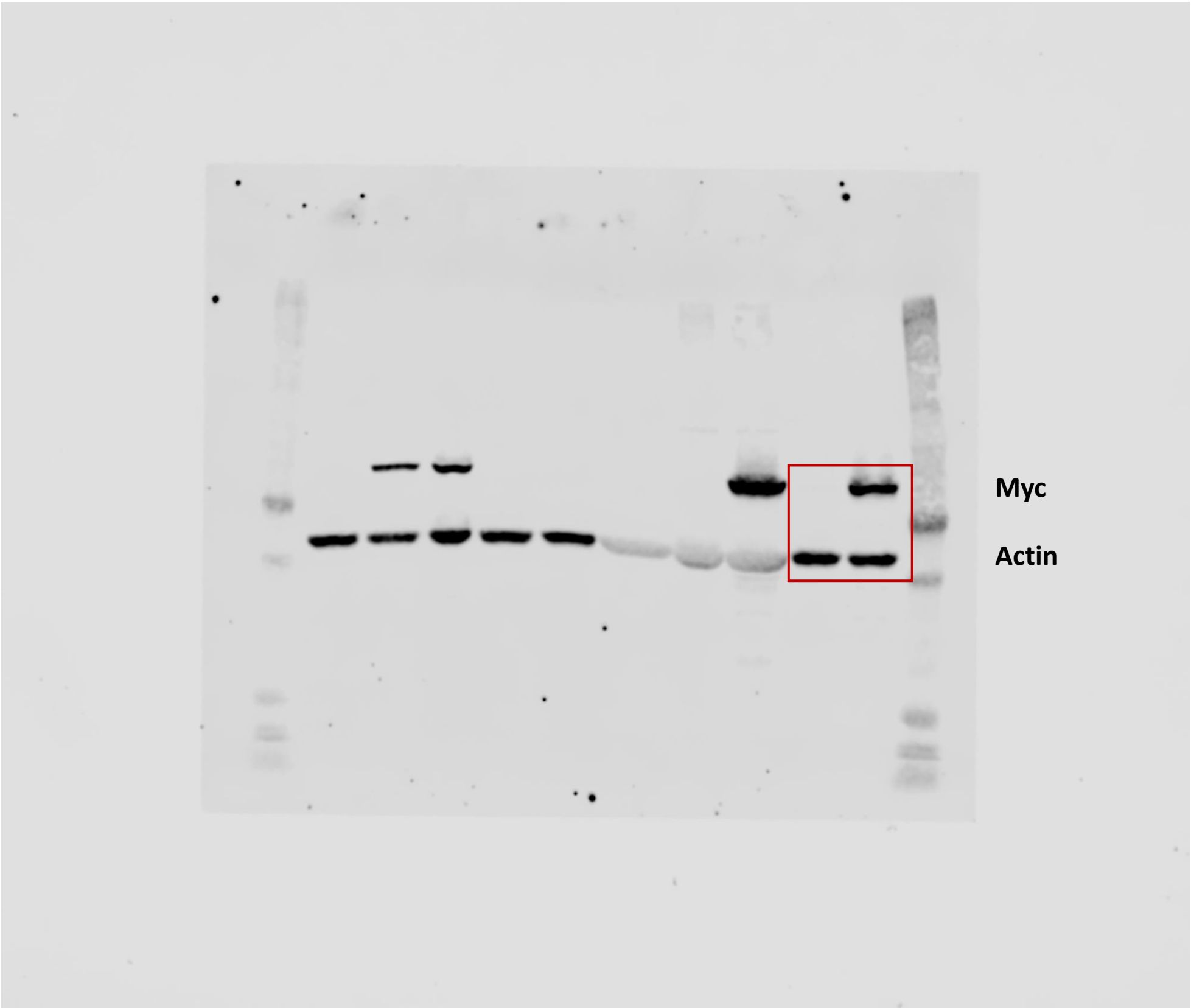

Supplement: Figure 2—figure supplement 1—source data 1. [file elife-87357-fig2-figsupp1-data1.zip › Figure 2 - Figure Supplement 1-source data 1/Figure 2 - Figure Supplement 1-source data 1.pdf]

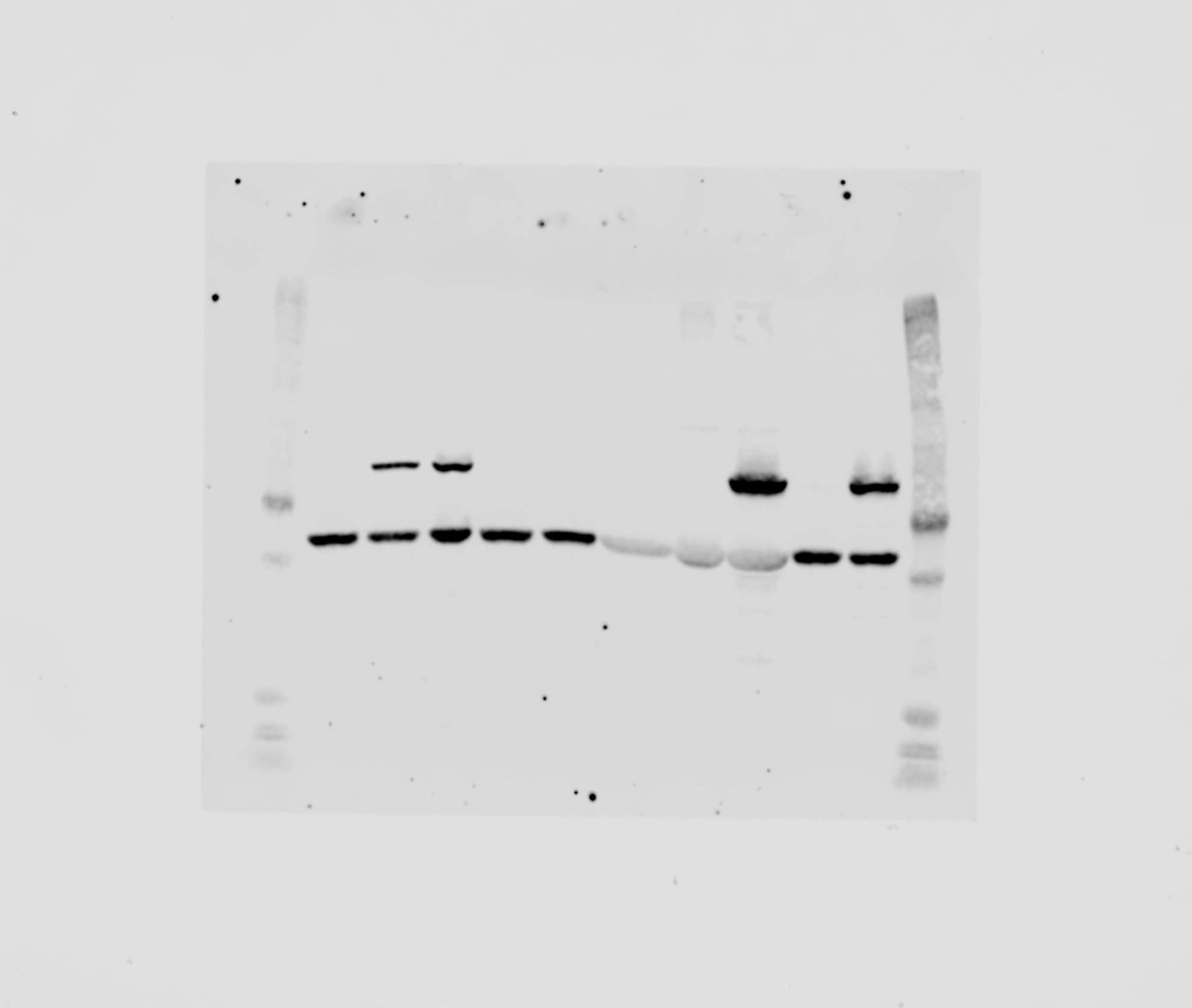

Supplement: Figure 2—figure supplement 1—source data 1. [file elife-87357-fig2-figsupp1-data1.zip › Figure 2 - Figure Supplement 1-source data 1/Image 1.tif]

Figure 3 -source data

A)

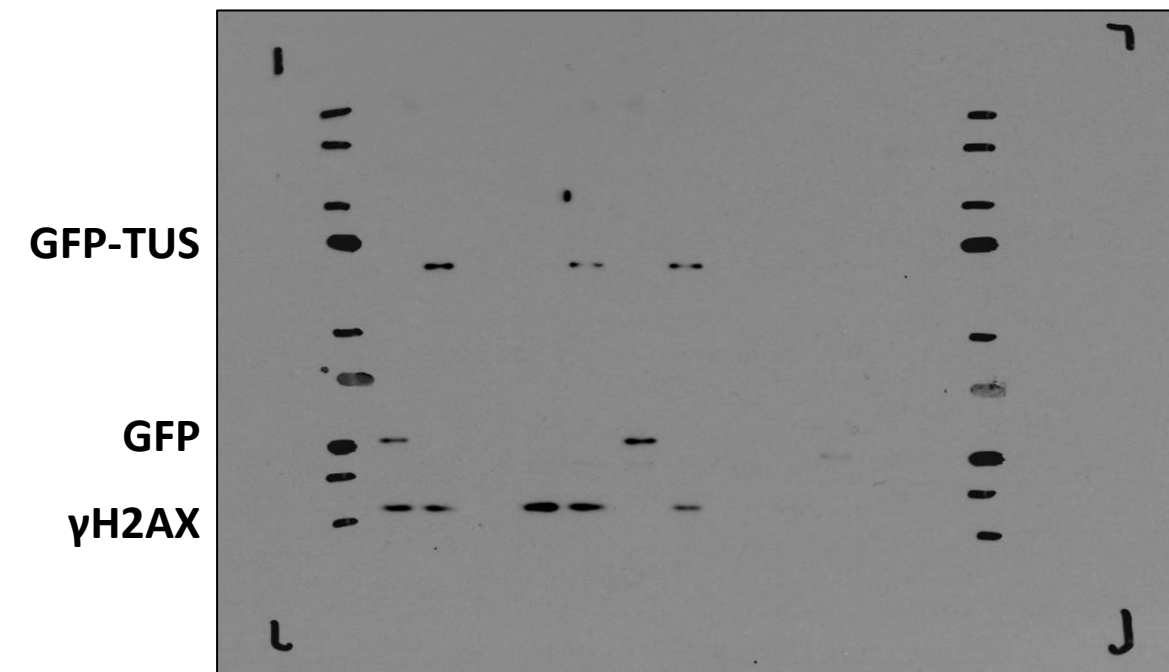

Supplement: Figure 3—source data 1. [file elife-87357-fig3-data1.zip › Figure 3 - source data 1/Figure 3 - source data 1.pdf]

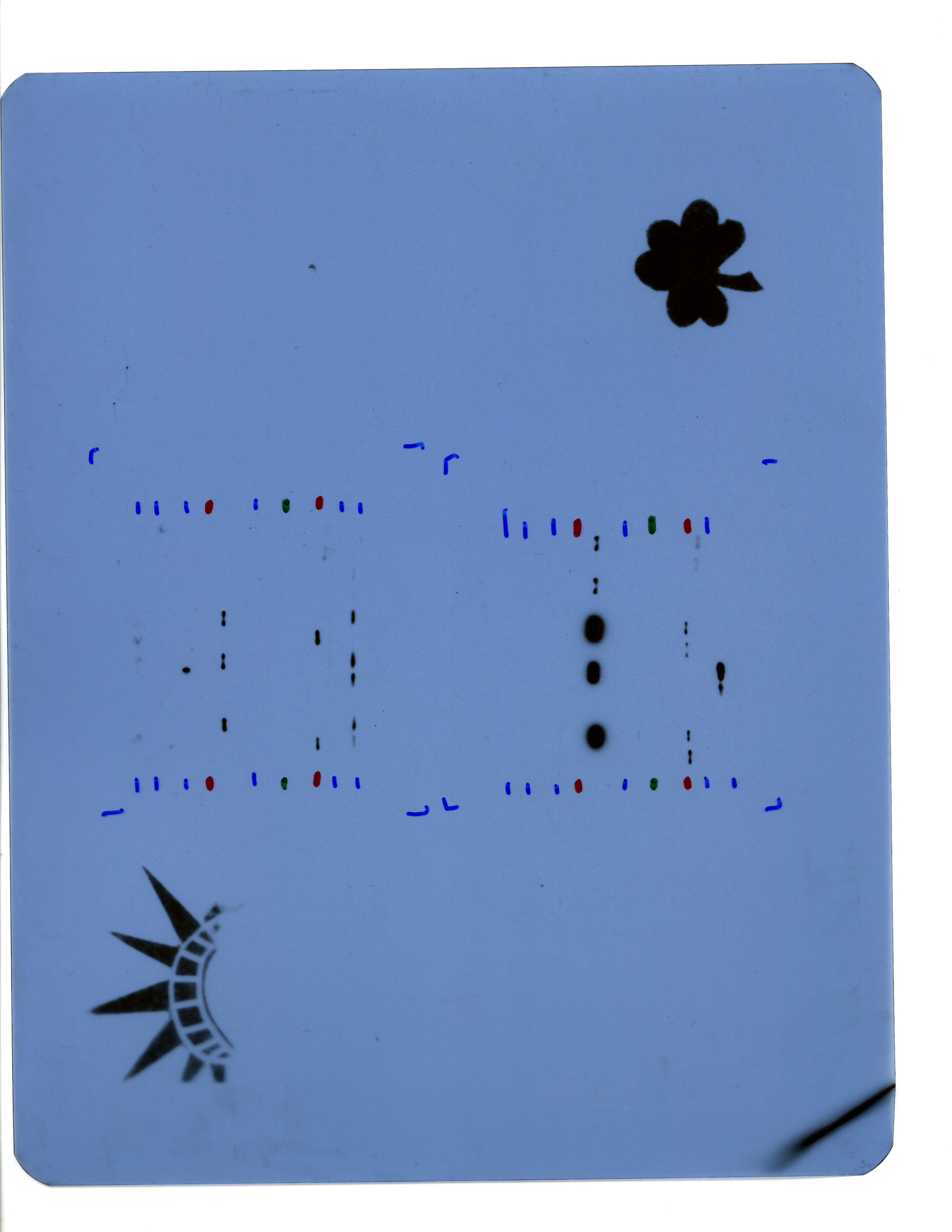

Supplement: Figure 3—source data 1. [file elife-87357-fig3-data1.zip › Figure 3 - source data 1/wblot 156117.tif]

Figure 3 - Figure supplement 1-source data

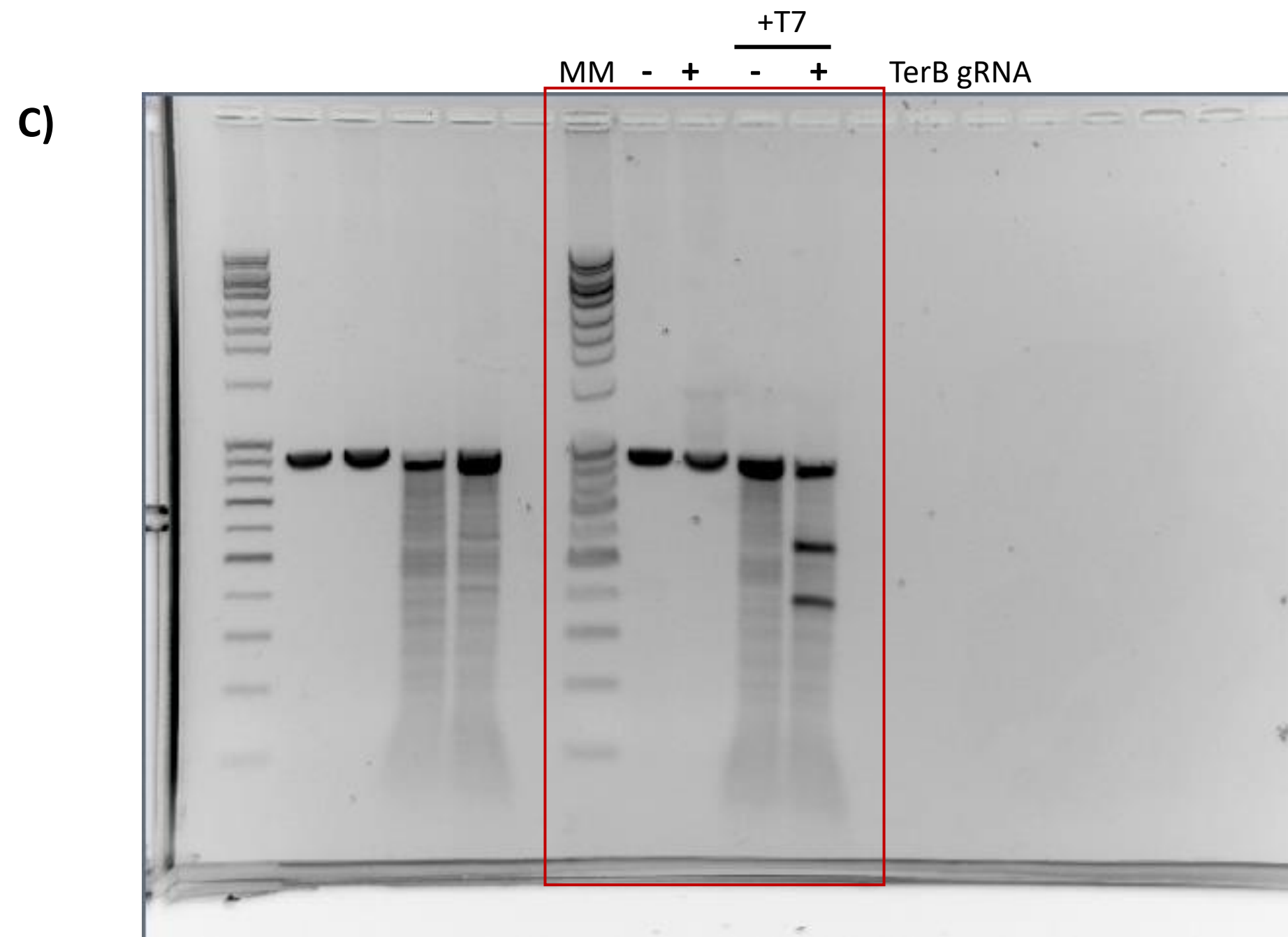

Supplement: Figure 3—figure supplement 1—source data 1. [file elife-87357-fig3-figsupp1-data1.zip › Figure 3 - Figure supplement 1-source data 1/Figure 3 - Figure supplement 1-source data 1.pdf]

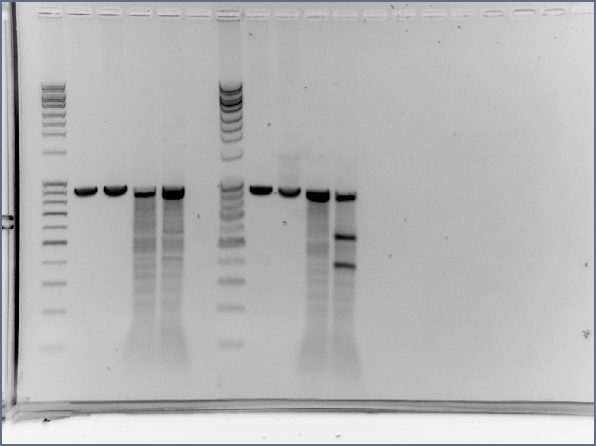

Supplement: Figure 3—figure supplement 1—source data 1. [file elife-87357-fig3-figsupp1-data1.zip › Figure 3 - Figure supplement 1-source data 1/Image 1.jpg]

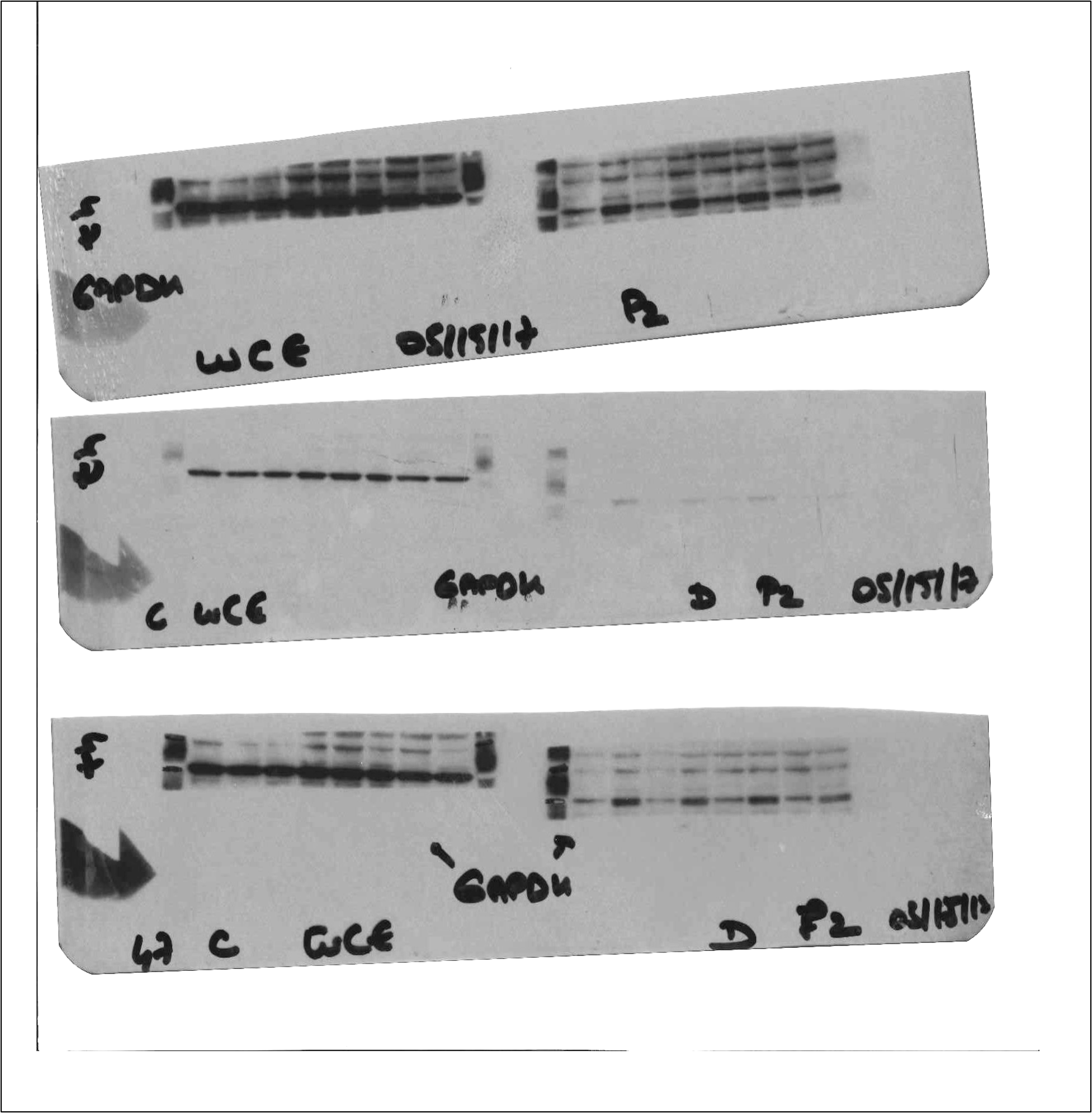

Supplement: Figure 4—source data 1. [file elife-87357-fig4-data1.zip › Figure 4 - source data 1/Fig 4A GAPDH.tif]

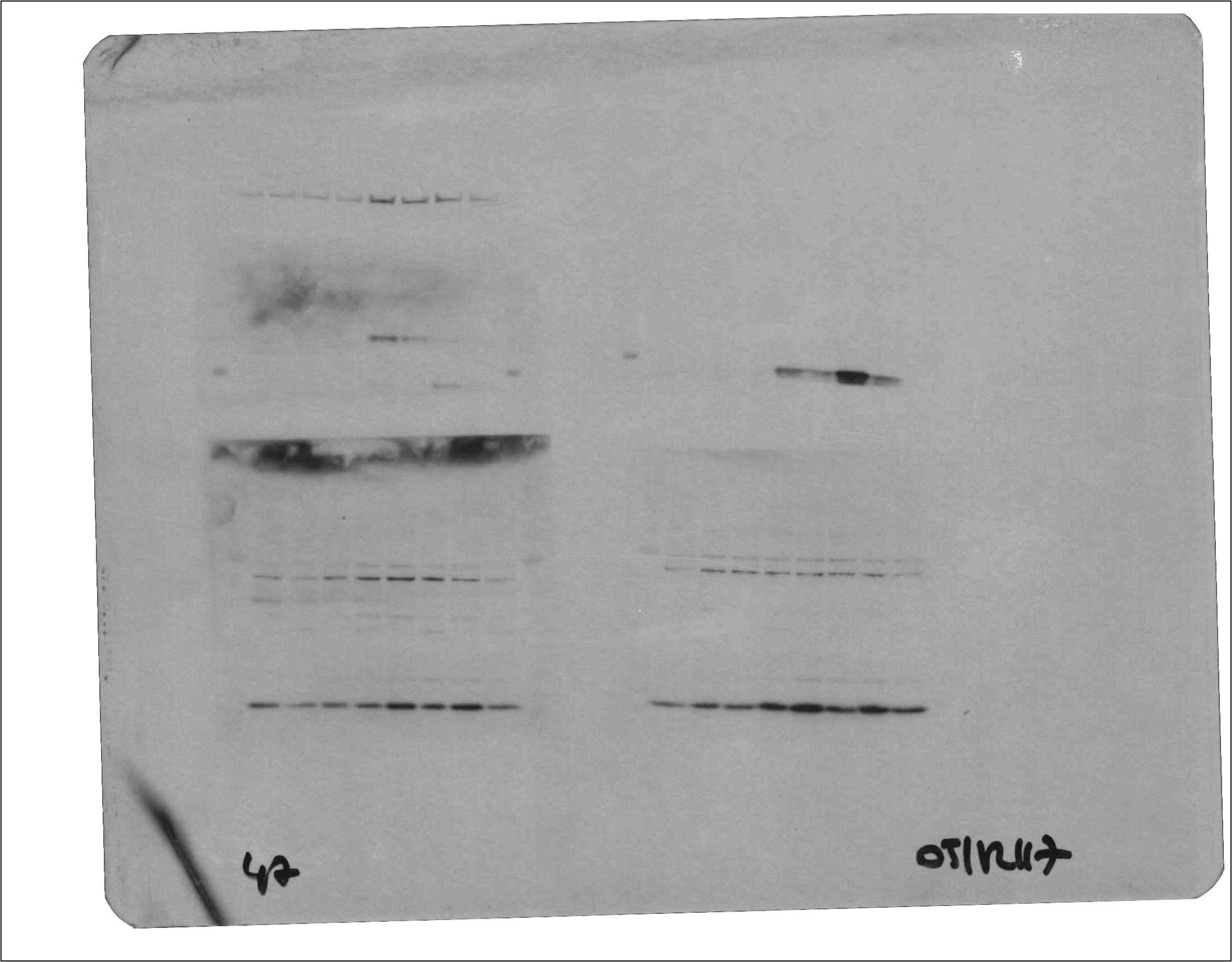

Supplement: Figure 4—source data 1. [file elife-87357-fig4-data1.zip › Figure 4 - source data 1/Fig 4A pATR (Th1989).tif]

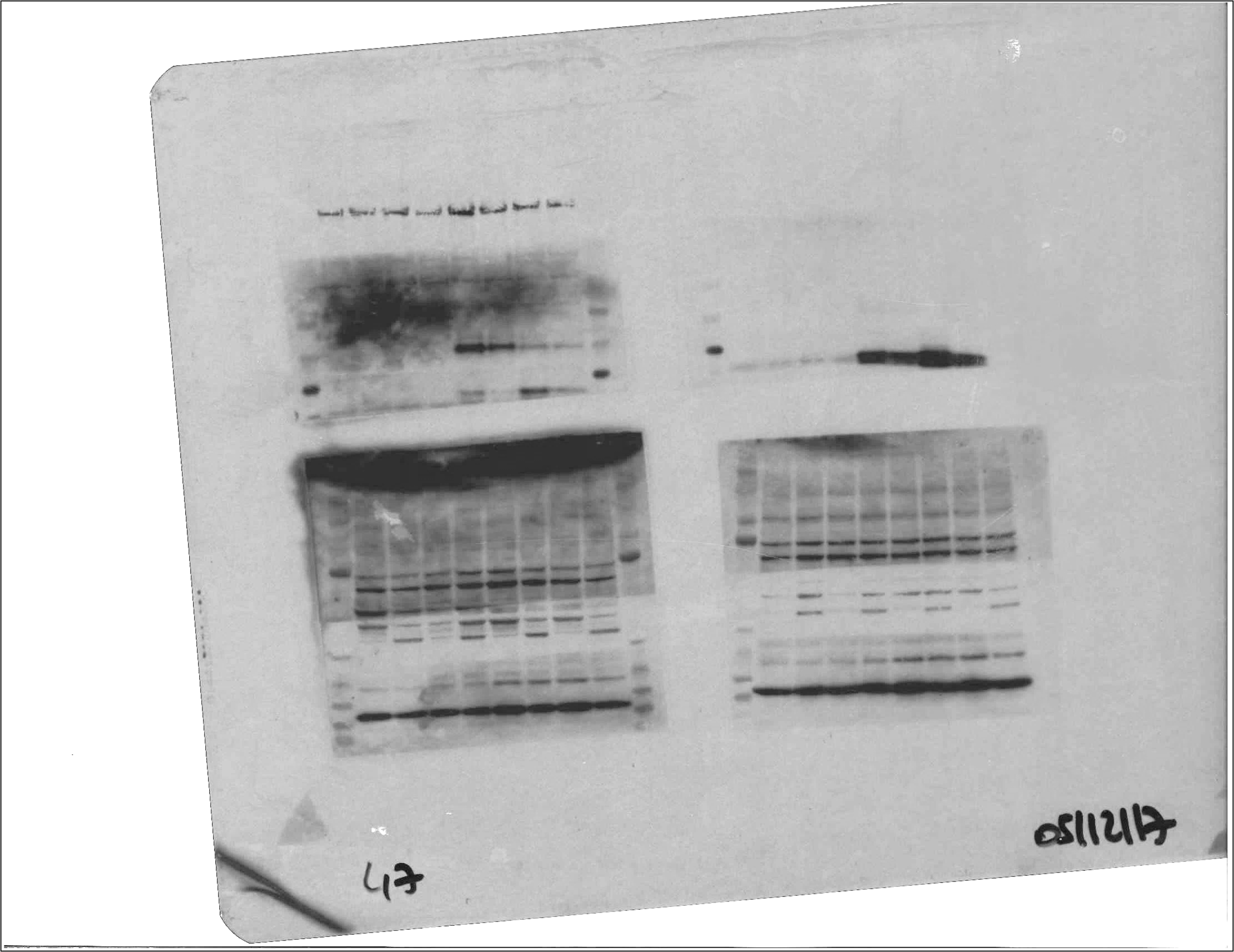

Supplement: Figure 4—source data 1. [file elife-87357-fig4-data1.zip › Figure 4 - source data 1/Fig 4A pCHK1 S345.tif]

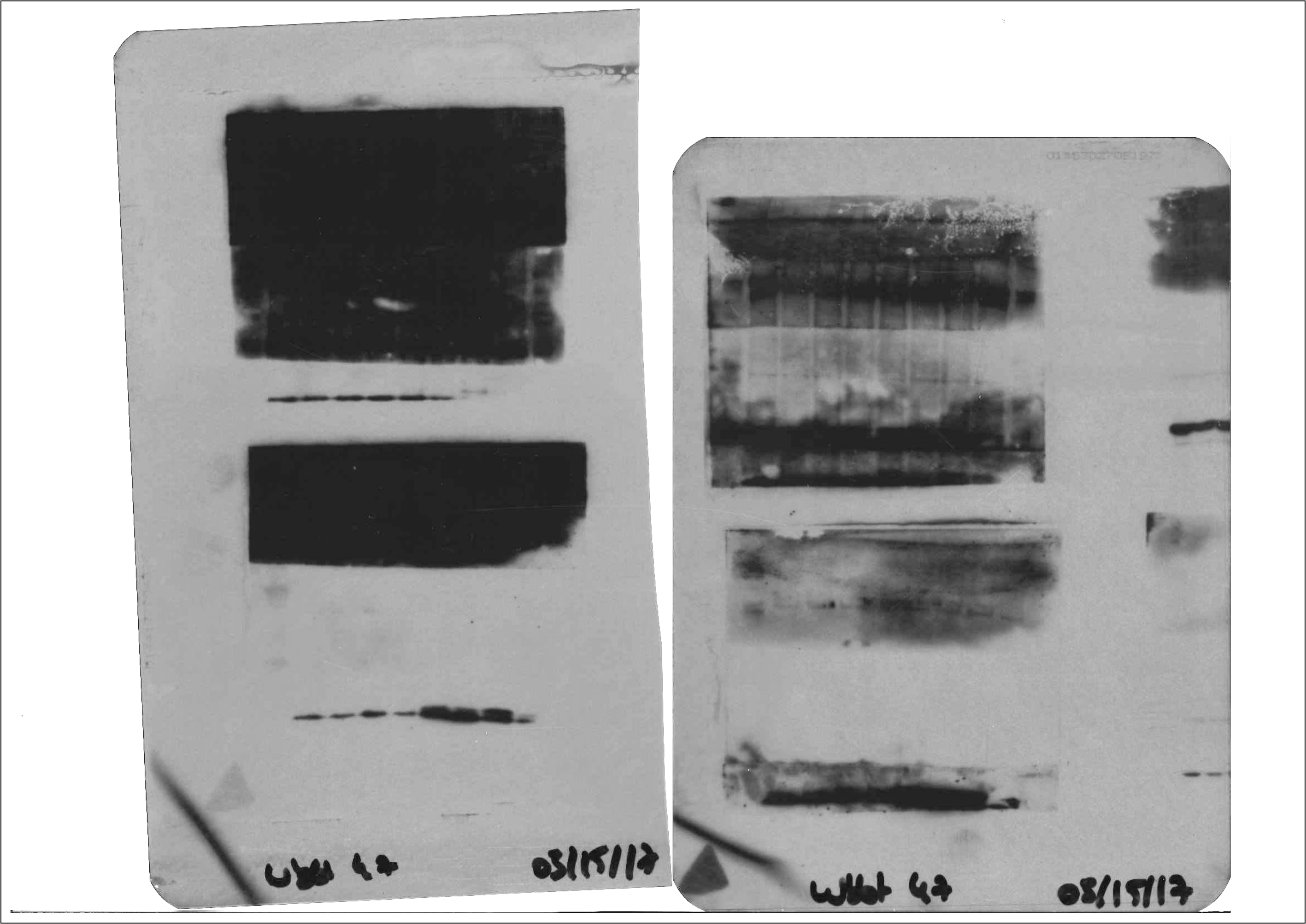

Supplement: Figure 4—source data 1. [file elife-87357-fig4-data1.zip › Figure 4 - source data 1/Fig 4A RPA.tif]

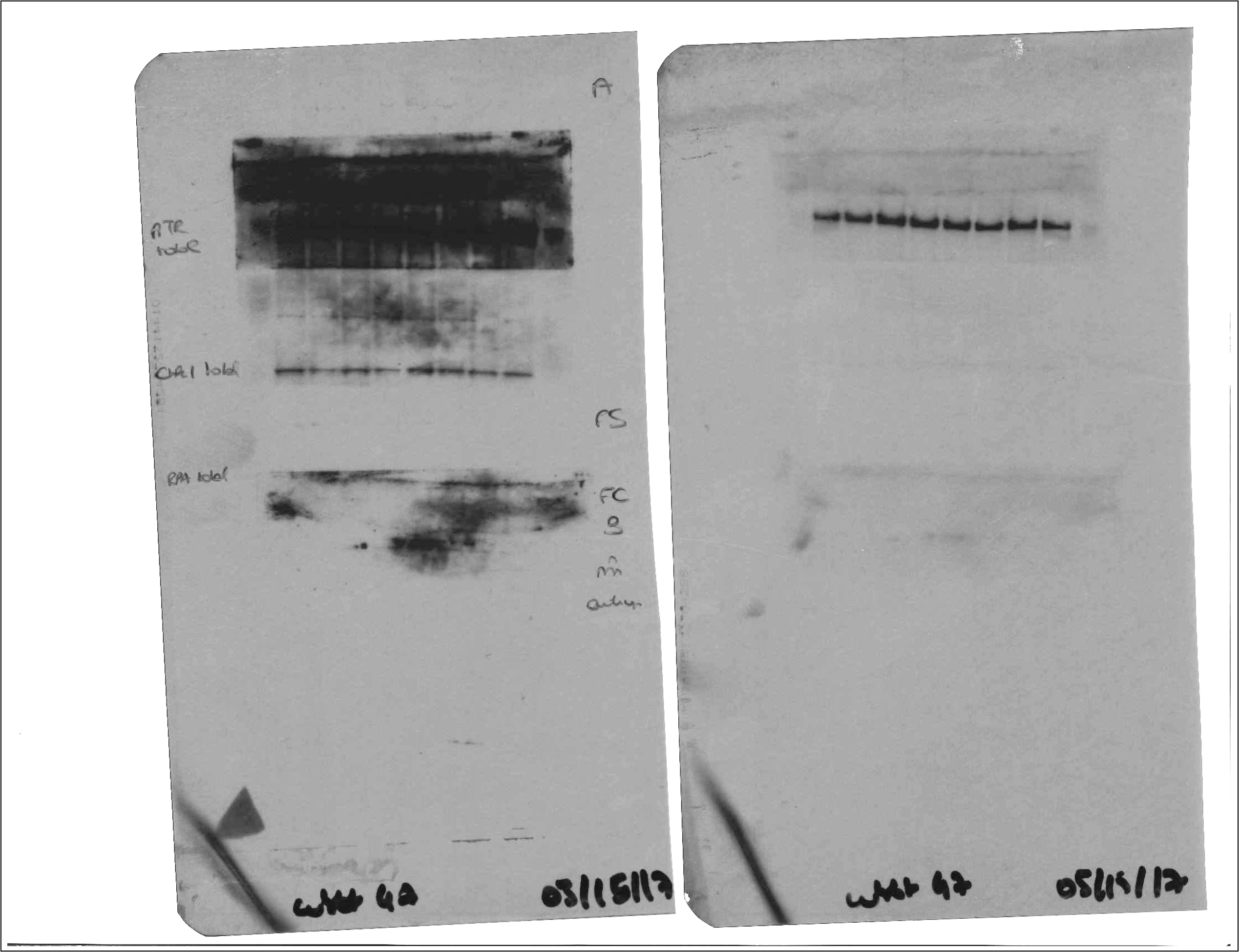

Supplement: Figure 4—source data 1. [file elife-87357-fig4-data1.zip › Figure 4 - source data 1/Fig 4A Total ATR.tif]

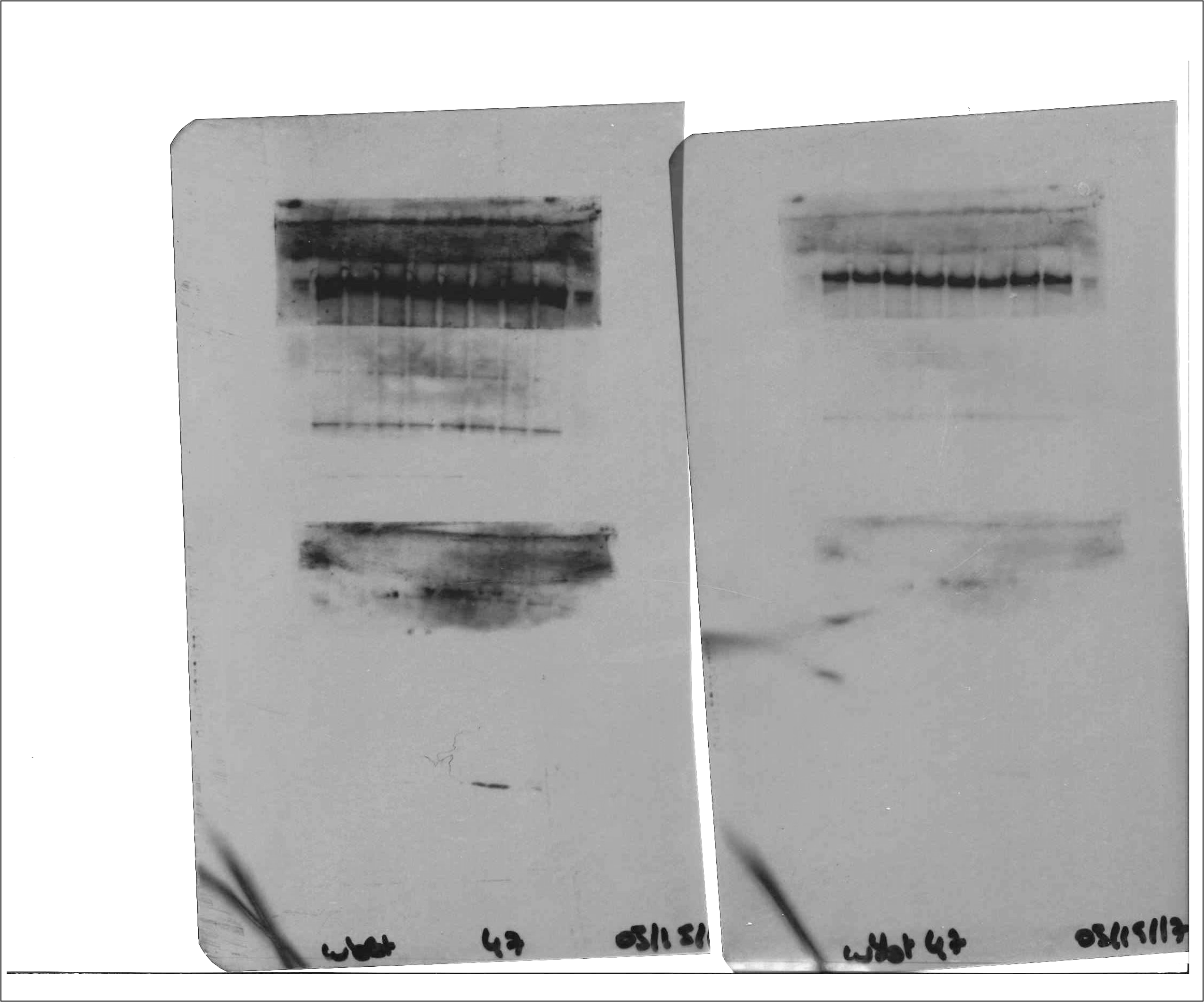

Supplement: Figure 4—source data 1. [file elife-87357-fig4-data1.zip › Figure 4 - source data 1/Fig 4A Total CHK1.tif]

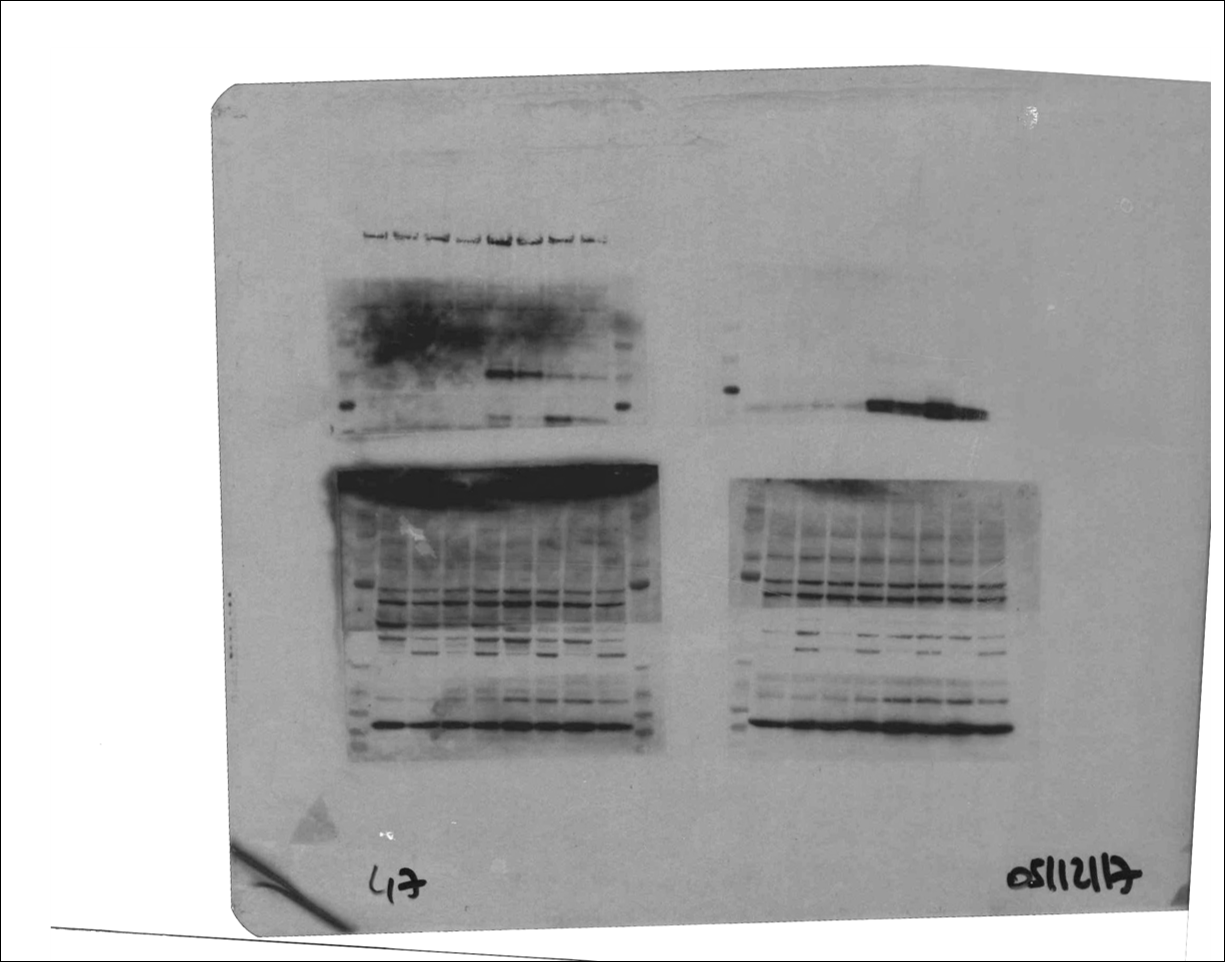

Supplement: Figure 4—source data 1. [file elife-87357-fig4-data1.zip › Figure 4 - source data 1/Fig 4A Tus.tif]

# Figure 4-source data

A)

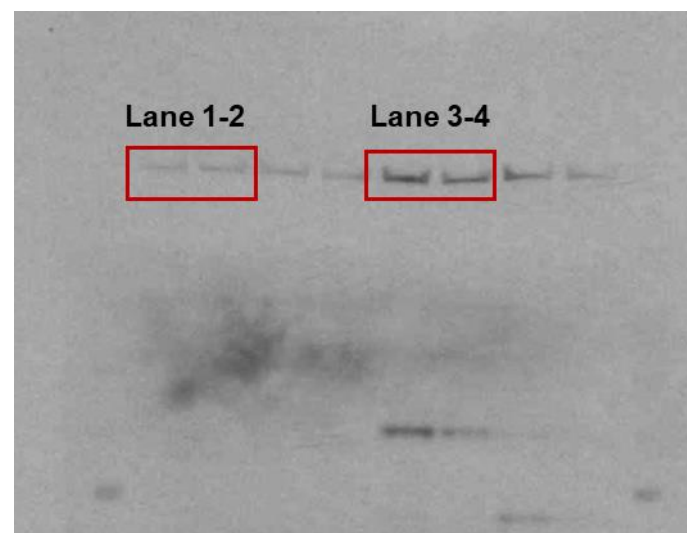

pATR (TH1989)

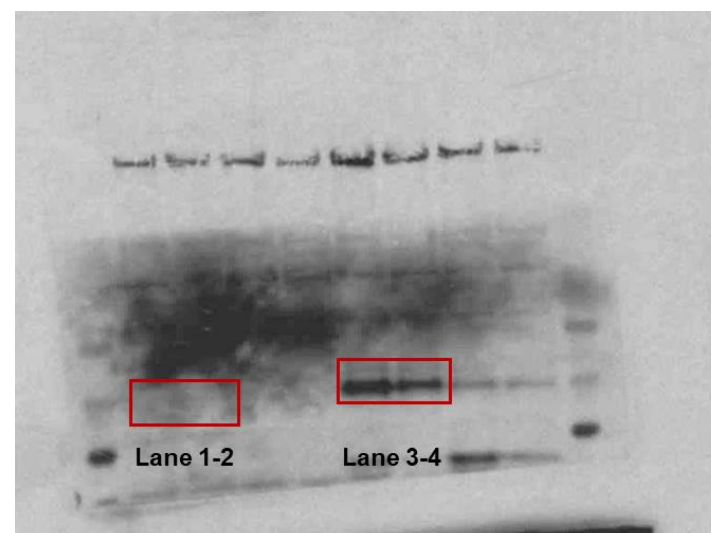

pCHK1 S345

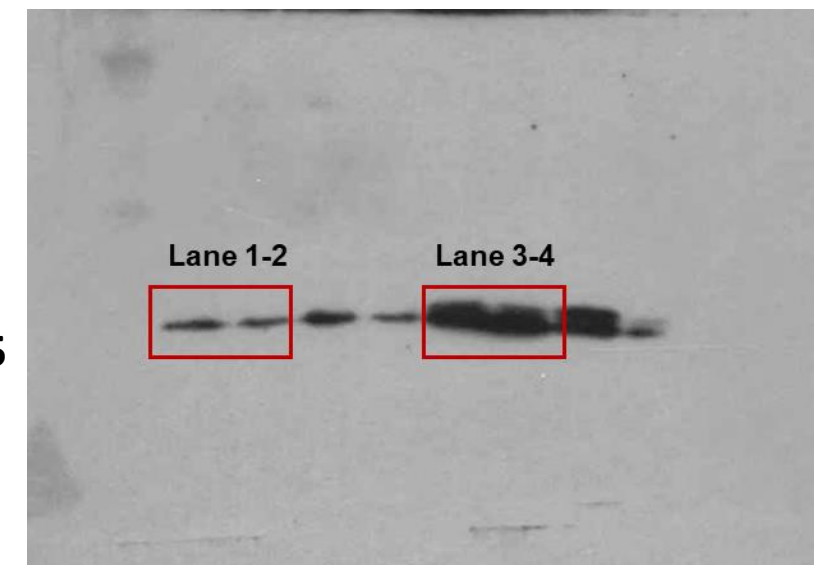

RPA/pRPA

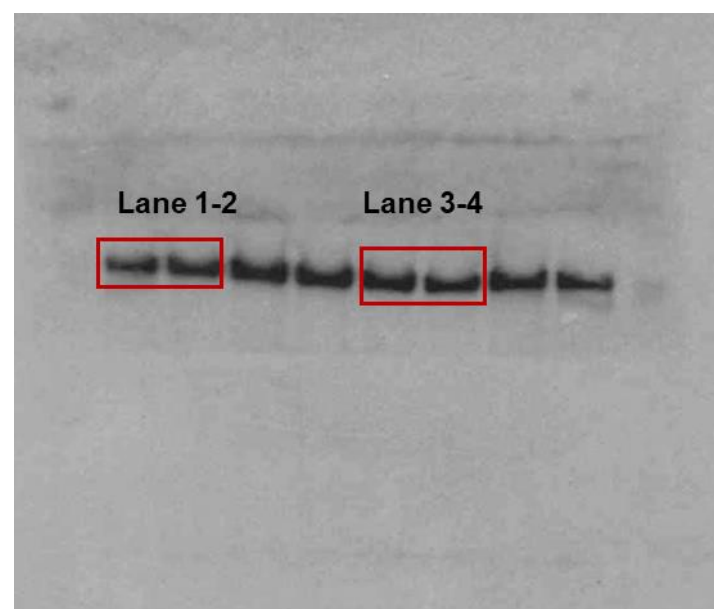

Total ATR

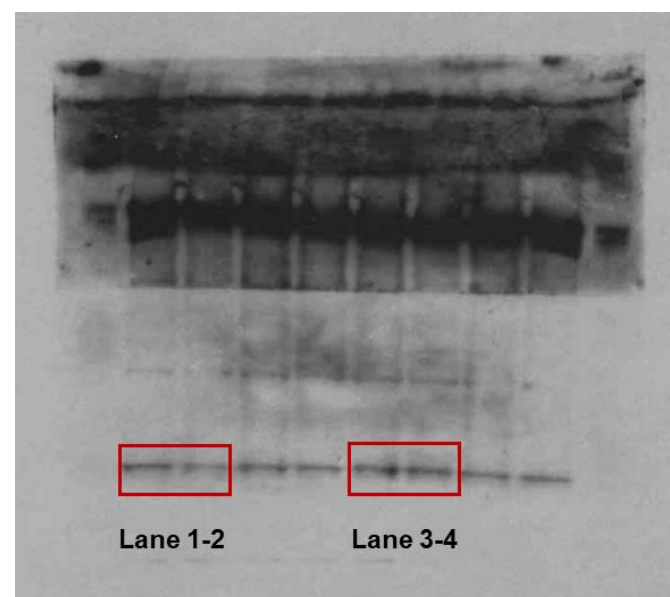

Total CHK1

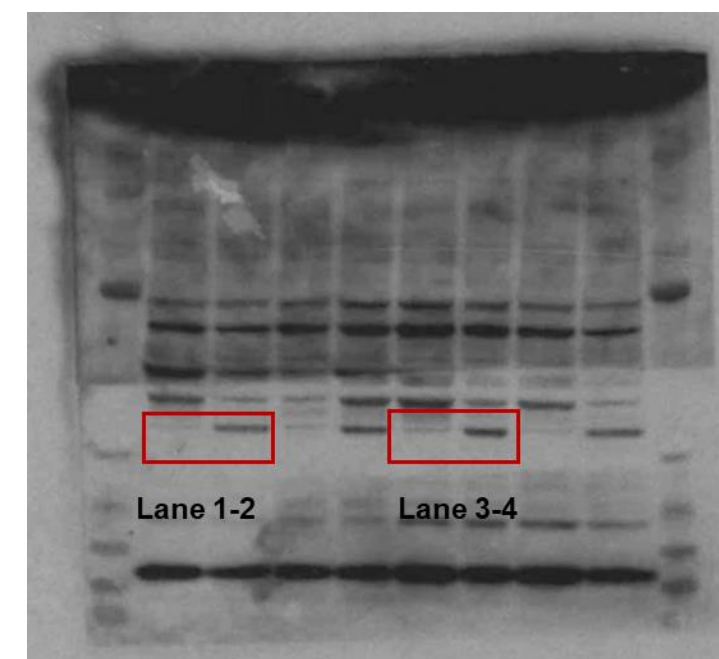

Tus

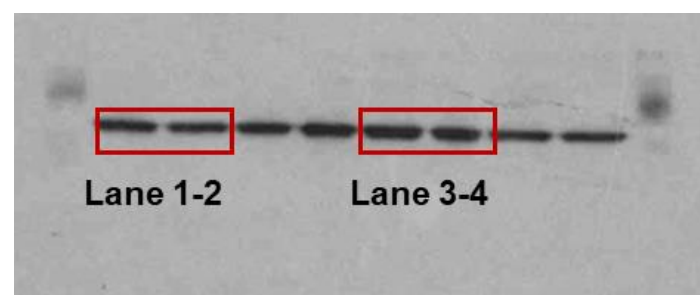

GAPDH

Supplement: Figure 4—source data 1. [file elife-87357-fig4-data1.zip › Figure 4 - source data 1/Figure 4 - source data 1.pdf]

Figure 4-source data

B)

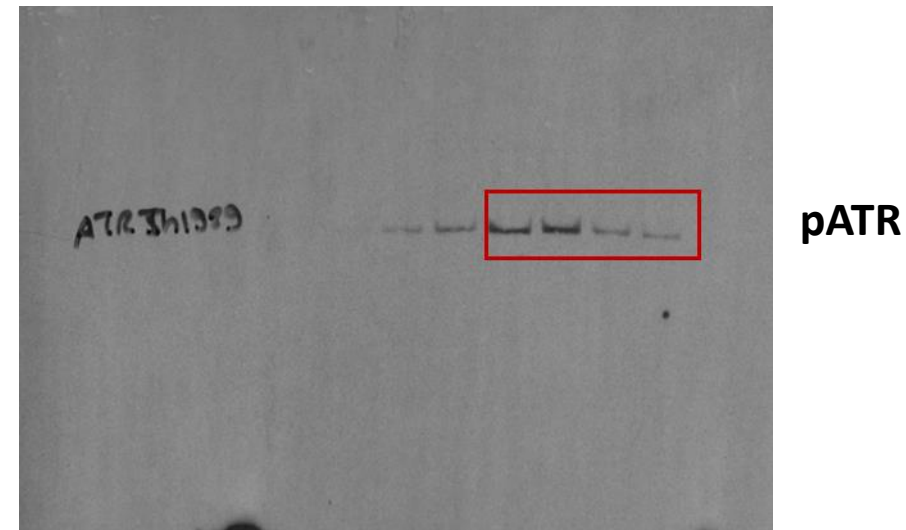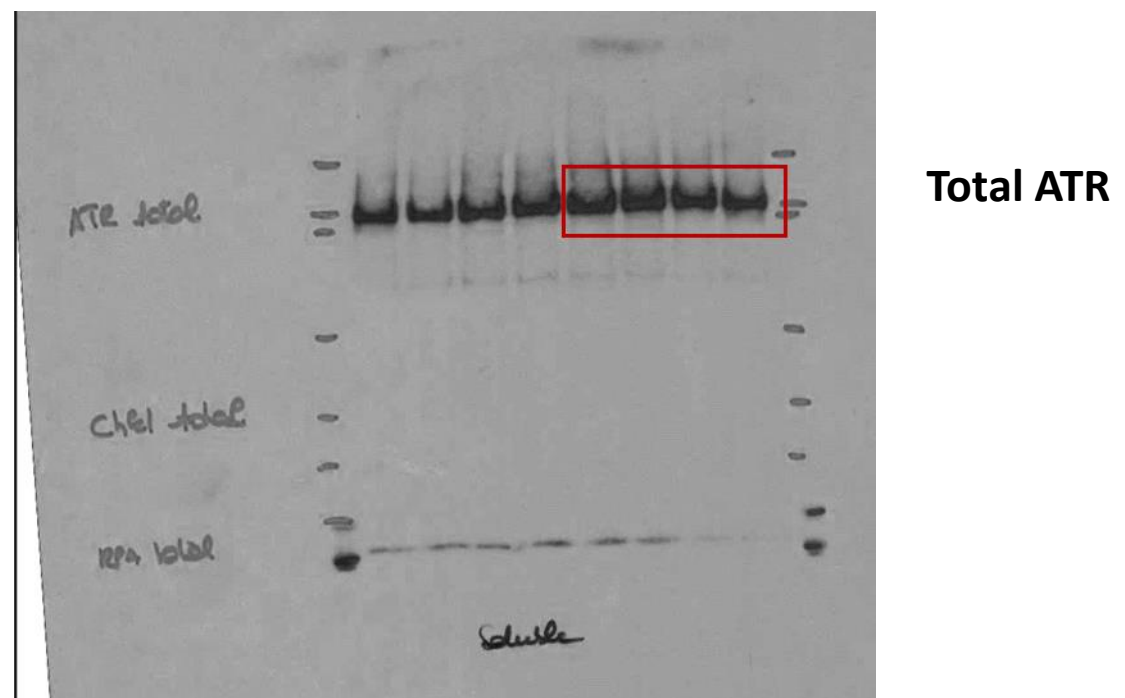

Supplement: Figure 4—source data 2. [file elife-87357-fig4-data2.zip › Figure 4 - source data 2/Figure 4 - source data 2.pdf]

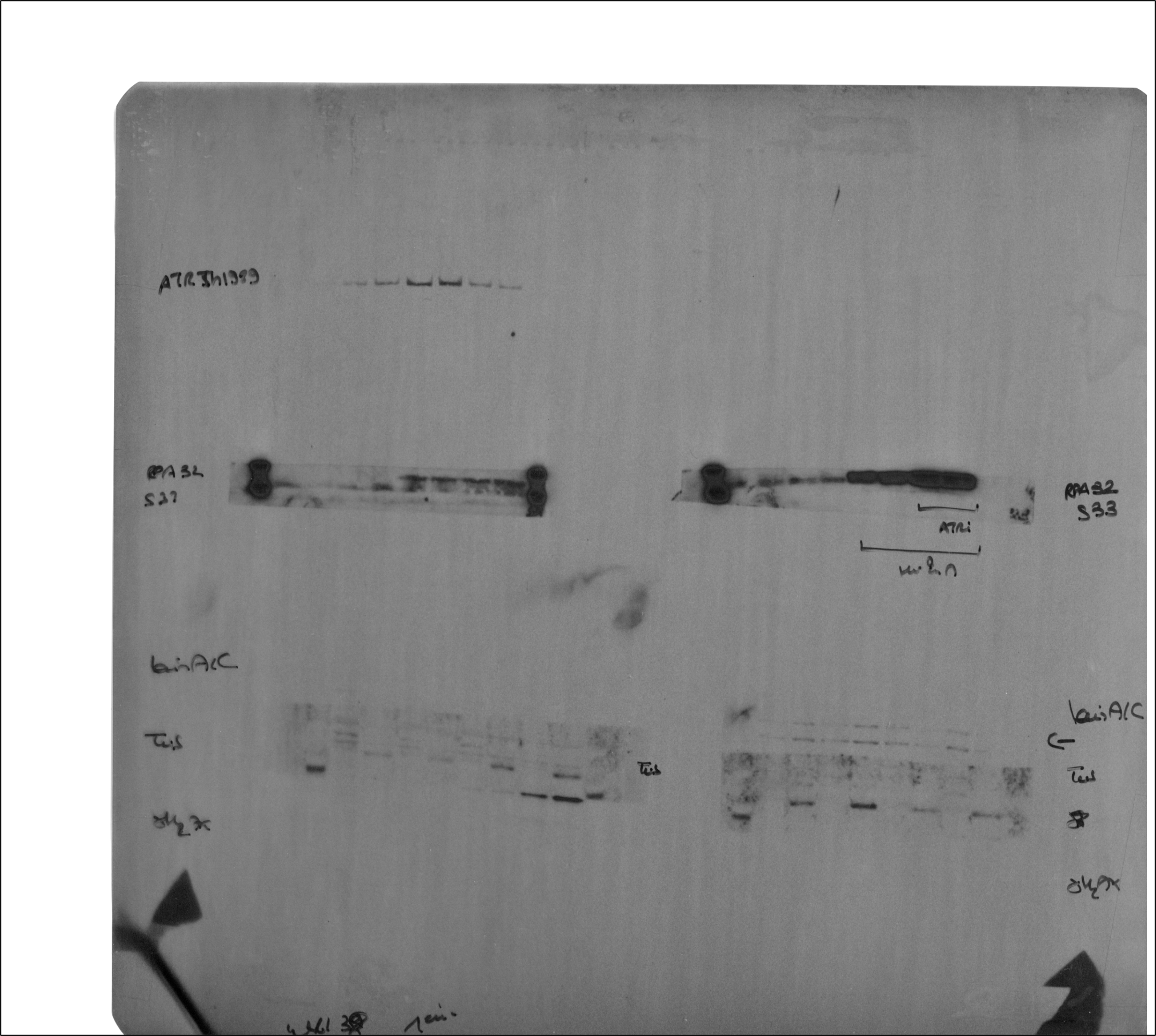

Supplement: Figure 4—source data 2. [file elife-87357-fig4-data2.zip › Figure 4 - source data 2/Figure 4B-pATR.tif]

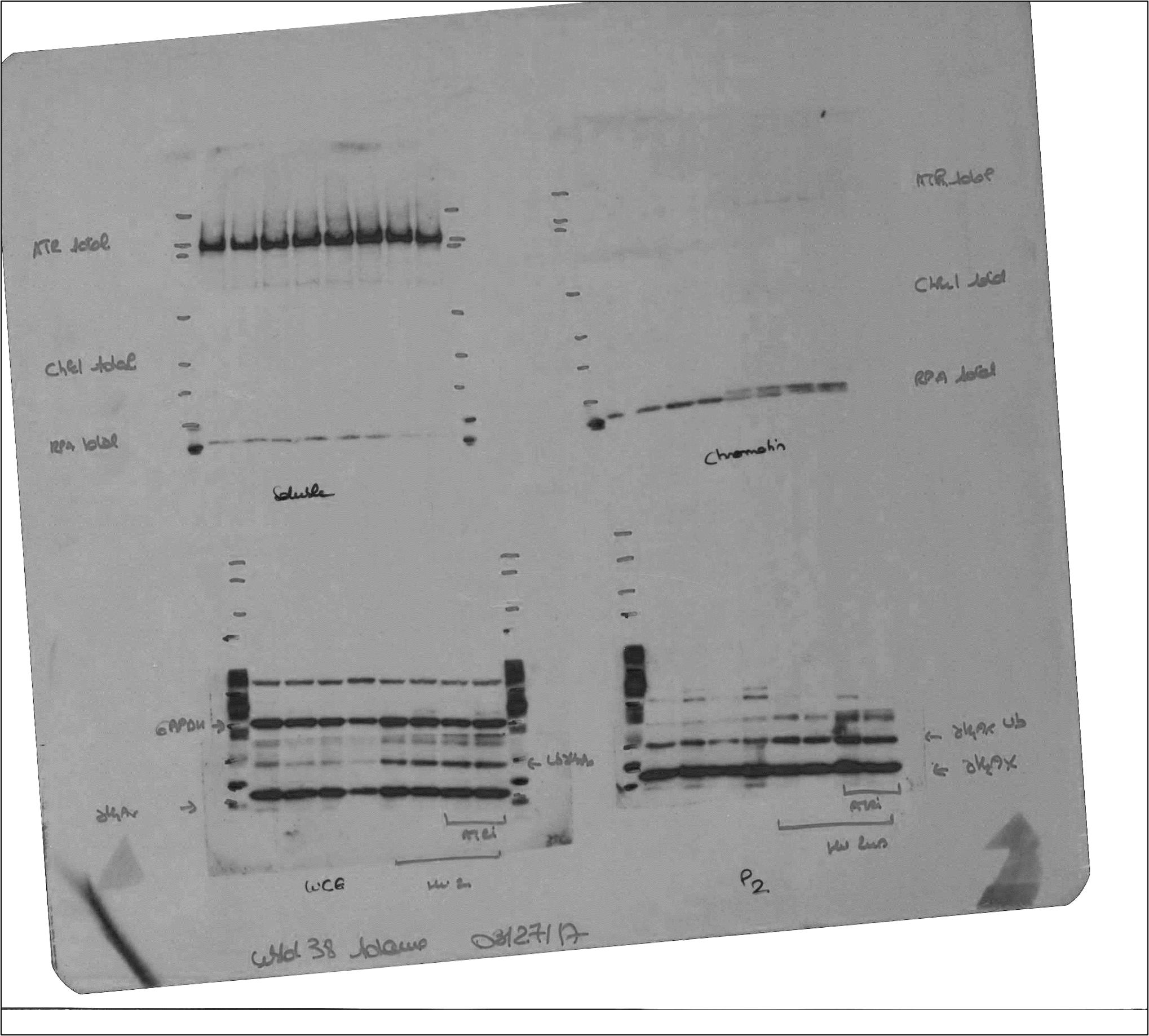

Supplement: Figure 4—source data 2. [file elife-87357-fig4-data2.zip › Figure 4 - source data 2/Figure 4B-Total ATR.tif]
