# Supplementary material for: A local ATR-dependent checkpoint pathway is activated by a site-specific replication fork block in human cells
Source: eLife. 2023 Aug 30;12:RP87357. doi: 10.7554/eLife.87357 (PMC10468204; doi:10.7554/eLife.87357)
Supplement: Supplementary file 1. — (a) Demonstration that the Tus-Ter replication fork block does not activate significant replication elsewhere in the genome. Replication characteristics of 200 kb global DNA segments that represent the total genome. These measurements do not include the segments containing the TerB sequence. The table compares MCF7 cells containing the TerB sequence with and without Tus induced. This was determined on stretched DNA molecules that had completely incorporated IdU, CldU, or a combination of both nucleotide analogs. (b) List of antibodies, oligos, and plasmids used in the study. [file elife-87357-supp1.docx]

|  | **MCF7 with integrated *TerB*** | **MCF7 with integrated *TerB* + Tus expressed** |
| --- | --- | --- |
| Total number of 200 kb DNA segments counted | 291 | 334 |
| Number of segments containing both red and green signal (RG) | 85 | 108 |
| Time to replicate (minutes) | 87 | 92 |
| Fork Rate (kb/min) per (RG) | 1.7 | 1.6 |

**Supplementary File 1a**

**Supplementary File 1b**

|  | **Manufacturer** | **Catalogue No.** |
| --- | --- | --- |
| **Antibodies** | | |
| Actin | Sigma | A2066 |
| Avidin, NeutrAvidin™, Alexa Fluor™ 350 conjugate | Invitrogen | A11236 |
| Anti-Mouse IgG, HRP-linked | Cell signaling | #7076 |
| Anti-Rabbit IgG, HRP-linked | Cell signaling | #7074 |
| ATR Th1989 | Genetex | GTX128145 |
| Chk1 S345 | Cell signaling | #2348 |
| FANCM | Abcam | Ab95014 |
| GAPDH FL-335 | Santa Cruz | sc-25778 |
| GFP | Abcam | Ab290 |
| GFP B-2 | Santa Cruz | SC 9996 |
| gH2AX s139 | Abcam | Ab2893 |
| gH2AX s139 | Abcam | Ab81299 |
| gH2AX s139 | Cell Signaling | 9718S |
| Goat anti-Rabbit, Alexa Fluor™ Plus 555 | ThermoFisher Scientific | A32732 |
| Goat anti-Rat IgG, Alexa Fluor™ 488 | Invitrogen | A-11006 |
| Goat anti-Mouse IgG, Alexa Fluor™ 568 | Invitrogen | A-11031 |
| Goat Anti-Mouse IRDye 680LT | LiCor | #926-68020 |
| Goat Anti-Rabbit IRDye 800CW | LiCor | #926-32211 |
| Goat Anti-Avidin D Antibody, Biotinylated | Vector Laboratories | BA-0300 |
| HA F-7 | Santa Cruz | sc-7392 |
| His | Abcam | Ab9108 |
| IgG | Cell signaling | #2729 |
| Lamin A/C | Santa Cruz | sc-6215 |
| MCM3 | Abcam | Ab4460 |
| Myc | Cell Signaling | #2276 |
| Purified Mouse Anti-BrdU | BD Biosciences | 347580 |
| Rat monoclonal anti-BrdU antibody | Abcam | ab6326 |
| RPA 32 S33 | Bethyl | A300-246 |
| Total ATR (N-19) | Santa Cruz | sc-1887 |
| Total Chk1 (G-4) | Santa Cruz | sc-8408 |
| Total RPA 32 | Cell Signaling | #2208 |
| Tus | This study | NA |
|  |  |  |
| **Oligonucleotides** | | |
| Primers for PCR amplification of pcDNA3-β-MYC-NLS-Tus,  Forward: AGTCGGTACCGAATTCGCCACCATGGAACAAAAGCTG  Reverse: AGTCGGCGGCCGCGCCGCTACCGTCAGCCACGTACAGGTGCA | This paper | N/A |
| Primers for PCR amplification of SNAP tag cDNA,  Forward: AGTCGCGGCCGCCGGCCACATGGACAAAGACTGCGAAATGAAGC  Reverse: ACTGCTCGAGTCAACCCAGCCCAGGCTTGC | This paper | N/A |
| sgRNA TerB1:TTGCGCTGCTTCGCGATGTA | This paper | N/A |
| Primer Pair PP-0-2 Forward: TCTGAGAATAGTGTATGCGG Reverse: AGATGCTGAAGATCAGTTGG | This paper | N/A |
| Primer Pair PP9  Forward: CGAGCTCGGATCAATAAGT  Reverse: AGAGTCGACCATAGGGGAT | This paper | N/A |
| Primer Pair PP2  Forward: AAAGTTCGAGTCTAGAGGGC  Reverse: GCATCAGAGCAGATTGTACT | This paper | N/A |
| Primer Pair PP52  Forward: TCCTACTTGGCAGTACATCT  Reverse: GGAAAGTCCCGTTGATTTTG | This paper | N/A |
| Primer Pair PP47  Forward: AGCGTTTAAACTTAAGCTTGGTA  Reverse: GGCCCTCTAGACTCGAAATAA | This paper | N/A |
| Primer Pair PP10  Forward: TTTAGGGTTCCGATTTAGTGCT  Reverse: ATTTTTTAACCAATAGGCCGA | This paper | N/A |
|  |  |  |
| **Plasmids** | | |
| Myc-NLS-TUS-SNAP | This study | N/A |
| pcDNA3-β-MYC-NLS-Tus | This study | N/A |
| pCMV3xnls | Lab stock | N/A |
| pCMV3xnls-HA | This study | N/A |
| pCMV3xnls-GFP | This study | N/A |
| pCMV3xnls-Tus | This study | N/A |
| pCMV3xnls-Tus-His | This study | N/A |
| pCMV3xnls-Tus-HA | This study | N/A |
| pCMV3xnls-Tus-GFP | This study | N/A |
| pWB15 | This study | N/A |
| pInd Tus-SNAP | This study | N/A |
| pInducer10L | Drosopoulos et al., Cell Reports 30 2020 | N/A |
| Fosmid | BACPAC Genomic | WI2-1478M20 |
|  |  |  |
